# Independent Predictors of 30-Day Readmission to Acute Psychiatric Wards in Patients With Mental Disorders: A Systematic Review and Meta-Analysis

**DOI:** 10.7759/cureus.42490

**Published:** 2023-07-26

**Authors:** Nazar Muhammad, Saifullah Talpur, Niraj Sangroula, FNU Washdave

**Affiliations:** 1 Psychiatry, Cornerstone Family Healthcare, Binghamton, USA; 2 Psychiatry, Liaquat National Hospital and Medical College, Karachi, PAK; 3 Psychiatry, College of Medical Sciences, Bharatpur, NPL; 4 Psychiatry, Children's Home of Wyoming Conference, Binghamton, USA

**Keywords:** systematic review and meta-analysis, acute psychiatric setting, factors, psychiatric disorder, readmission

## Abstract

Psychiatric readmissions have long been considered significant indicators for healthcare planning. The aim of this study was to identify factors influencing early (30-day) readmissions to acute psychiatric wards. A meta-analysis and systematic review were conducted according to Meta-analysis of Observational Studies in Epidemiology (MOOSE) guidelines. Comprehensive database searching was conducted using online databases, including PubMed and Google Scholar, to search for articles identifying factors associated with early (30-day) readmissions to acute psychiatric wards. Keywords used to search for relevant articles included "Mental illness," "readmission," and factors along with their synonyms and Medical Subject Headings (MeSH) terms. The search included studies published between 2011 and June 2023. A total of 13 studies were included in this meta-analysis. The pooled rate of the 30-day readmission was 16% (95% confidence interval: 13%-20%). A pooled analysis showed that factors significantly associated with an unplanned hospital readmission included gender, length of stay, and insurance status as predictors of the unplanned hospital readmission among individuals with psychiatric illness. Additionally, we also found that the rate of 30-day unplanned admissions was greater in patients with schizophrenia, followed by personality disorder, bipolar disorder, depression, and substance use. This study highlights the importance of providing targeted interventions and support for individuals with these conditions to reduce the risk of readmissions.

## Introduction and background

In the era of healthcare reform, there has been a growing concern among health insurance providers, policymakers, and healthcare professionals regarding the frequent readmission of patients after being discharged from hospitals [1]. While the focus has primarily been on readmissions to general hospitals for short-term acute care, there is increasing interest in understanding the readmission rates following psychiatric hospitalization. Psychiatric readmissions have long been considered significant indicators for healthcare planning due to their impact on the quality and continuity of patient care, as well as the substantial costs associated with additional inpatient treatment [2-3]. The rate at which psychiatric patients are readmitted within 30 days after discharge serves as an established measure to evaluate the performance of behavioral health systems [4]. It is also linked to the quality of inpatient hospital care and the availability of community-based aftercare services [5]. While previous studies have focused on 30-day psychiatric readmissions, there has been a lack of studies examining the specific influences of patient and treatment factors during different time frames within this 30-day period [6-7].

In various research studies, the documented rates of psychiatric readmissions span from 10% during a one-month period following discharge to as high as 86% within a seven-year timeframe [8-9]. Furthermore, there is evidence indicating a higher probability of readmissions associated with several factors. These factors include predisposing elements such as a previous history of psychiatric hospitalization [10-11], the severity of the illness, the use of alcohol and substances [12], and a lower level of patient functioning at the time of discharge [13]. Additionally, aftercare-related factors, such as inadequate community support and insufficient ambulatory care visits [14], contribute to the likelihood of a readmission. Overall, readmissions that occur shortly after discharge have been recognized as valuable indicators strongly linked to the quality of care provided by hospitals [13].

Regardless of the method used to assess readmissions, the rates of readmission specifically related to mental illnesses varied from 5% [15] to 43% [16], which is higher compared to the readmission rates associated with general health conditions ranging from 2.8% to 38% [17]. Various efforts have been undertaken to decrease readmissions, particularly by identifying patients who are at higher risk of being readmitted after being discharged from their initial admission. Recognizing risk factors for readmission is widely acknowledged as crucial, as it enables clinicians to identify individuals who may be susceptible to such occurrences. By identifying these factors, clinicians can then take steps to mitigate them, thereby reducing the burden on the individuals using these services, their families, and the healthcare system. While readmission during the course of a chronic mental illness is not unexpected, it is typically unexpected, particularly within a short timeframe (within 30 days) after discharge, especially if the individual is already engaged with mental health outpatient services. Therefore, this study was conducted to identify factors influencing early (30-day) readmission to acute psychiatric wards.

## Review

Methodology

The current meta-analysis and systematic review were performed according to Meta-analysis of Observational Studies in Epidemiology (MOOSE) guidelines. Comprehensive database searching was conducted using online databases, including PubMed, Web of Science and Google Scholar, to search for articles identifying factors associated with early (30-day) readmission to acute psychiatric wards. Keywords used to search for relevant articles included "Mental illness," "readmission," and factors along with their synonyms and Medical Subject Headings (MeSH) terms.

The search included studies published between 2011 and June 2023. Only studies published in the English language were included in this study. Full articles of potentially appropriate abstracts were reviewed after removing duplicates. The full text of eligible studies was obtained, and a detailed assessment was done based on predefined inclusion and exclusion criteria. The reference lists of all included articles were manually searched. Searching and selection of studies were done independently by two authors. Any disagreement between the two authors was resolved through consensus.

Study Inclusion and Quality Assessment

We included studies, published from 2011 onwards, that assessed factors associated with 30-day readmission to acute psychiatric wards due to any psychological issues. Reviews and letters to editors, and studies published in languages other than English were excluded. Quality assessment of included studies was performed independently by two authors using the Newcastle-Ottawa Scale. The Newcastle-Ottawa Scale evaluates three main domains of study quality: selection of study groups, comparability of groups, and ascertainment of either the exposure or outcome of interest. Each domain is further divided into specific criteria that are assessed to assign a score to each study. The scores are then used to judge the overall quality of the study and its suitability for inclusion in a meta-analysis. Any disagreement between the two authors in the process of quality assessment was resolved through discussion.

Data Extraction and Data Analysis

Data were extracted from the included studies using a pre-designed spreadsheet developed on Microsoft Excel (Microsoft, Redmond, WA). The extracted data included the first author's name, year of publication, region where the study was conducted, sample size, number of 30-day readmissions, and details about the factors associated with 30-day readmission.

Data were analyzed using RevMan, version 5.4.1 (Cochrane Collaboration, London) and Stata, version 16.0 (StataCorp LLC, College Station, TX). To determine factors associated with 30-day readmission, odds ratios (ORs) were calculated with 95% confidence intervals (CIs) for categorical variables, and mean differences (MDs) with 95% CIs for continuous variables. A p-value less than 0.05 was considered significant. To account for heterogeneity among studies, a random-effects model was employed for all analyses. We compared rates of 30-day readmission among five illnesses that included depressive disorder, bipolar disorder, substance abuse, schizophrenia, and personality disorder using network meta-analysis; pairwise comparisons were done and presented in the form of forest plot. We rank conditions with high risk of 30-day readmission using the Surface Under the Cumulative Ranking (SUCRA) score. The presence of statistical heterogeneity was evaluated using both the Cochran Q test and the Higgins I-square test. The Higgins I-square test categorized heterogeneity as follows: I-square <25% denoted low heterogeneity, 25%-50% denoted moderate heterogeneity, and values exceeding 50% indicated severe heterogeneity.

Results

**Figure 1:**
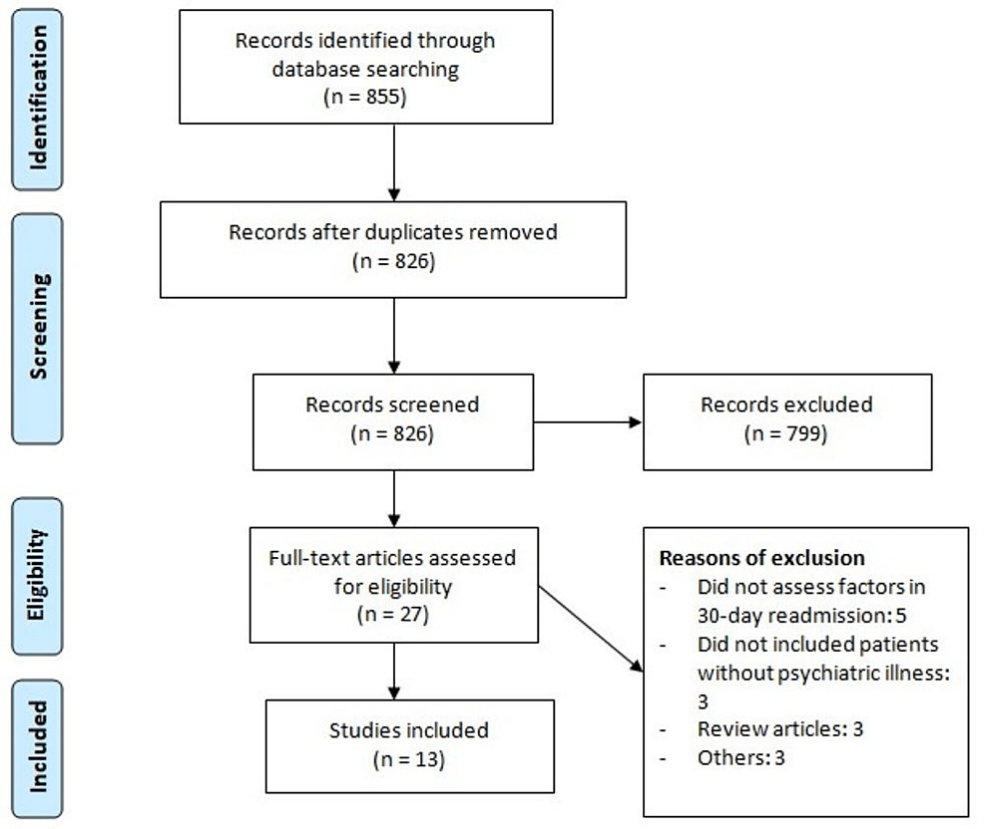
Study selection process

Figure 1 shows the Preferred Reporting Items for Systematic Reviews and Meta-analyses (PRISMA) flowchart of study selection. Online database searching generated 855 records. After removing duplicates, 826 records were initially screened using their titles and abstracts. Based on initial screening, 27 records were passed through detailed assessment. Finally, 13 studies were included in this meta-analysis. Table 1 shows the characteristics of included studies. Majority of studies were conducted in the United States. The pooled rate of 30-day readmission was 16% (95% CI: 13%-20%). Table 2 shows quality assessment of included studies.

**Table 1:** Characteristics of included studies

Author name	Year	Region	Disorder	Sample size	Number of readmissions
Becker et al. [18]	2016	United States	All psychiatric illnesses	1,689,797	314,742
Boaz et al. [19]	2013	United States	Schizophrenia	5557	2767
Chen et al. [20]	2018	United States	Mood disorders and substance abuse	296,912	31,096
Cook et al. [21]	2020	United States	Schizophrenia, bipolar disorder, and major depressive disorder	43,817	5932
Everett et al. [22]	2022	United States	Schizophrenia, bipolar disorder, and major depressive disorder	1034	197
Del Favero et al. [23]	2020	Italy	All psychiatric illnesses	798	128
Han et al. [24]	2020	China	All psychiatric illnesses	7224	1289
Hariman et al. [25]	2020	Hong Kong	Psychotic spectrum disorders	30,707	2178
Lorine et al. [26]	2015	United States	All psychiatric illnesses	207	62
Ortiz et al. [27]	2019	United States	All psychiatric illnesses	60,254	4829
Roque et al. [28]	2017	United States	All psychiatric illnesses	1152	52
Vigod et al. [29]	2015	Canada	All psychiatric illnesses	32,769	3022
Zhu et al. [30]	2022	China	Major depressive disorder	12,976	431

**Table 2:** Quality assessment of included studies

Author name	Selection	Exposure	Outcome	Overall
Becker et al. [18]	3	2	3	Good
Boaz et al. [19]	2	2	3	Good
Chen et al. [20]	2	1	3	Fair
Cook et al. [21]	3	2	2	Good
Everett et al. [22]	3	2	2	Good
Del Favero et al. [23]	3	1	2	Fair
Han et al. [24]	2	2	3	Good
Hariman et al. [25]	2	2	3	Good
Lorine et al. [26]	3	1	2	Fair
Ortiz et al. [27]	3	2	2	Good
Roque et al. [28]	2	2	2	Good
Vigod et al. [29]	3	2	3	Good
Zhu et al. [30]	3	2	2	Good

Factors Associated With 30-Day Readmission

Gender: Thirteen studies were included in the pooled analysis of impact of gender on 30-day readmission. As shown in Table 3, the odds of males were significantly higher in patients who were readmitted compared to patients who were not readmitted (OR: 1.15, 95% CI: 1.07-1.24; p-value: 0.001). High heterogeneity was reported among the study results (I-square: 95%).

**Table 3:** Factors associated with 30-day readmission OR: odds ratio; CI: confidence interval ^Presented as mean difference (95% CI) *Significant at p<0.05

Factors	OR	95% CI	I-square
Gender (male)	1.15	1.07, 1.24*	95%
Marital status (unmarried)	1.52	1.21, 1.91*	91%
Employment status (unemployed)	1.21	0.94, 1.56	54%
Insurance status (uninsured)	0.65	0.53, 0.80*	95%
Age (years)^	-0.55	-2.5, 1.39	99%
Length of stay (days)^	-1.18	-2.16, -0.19*	86%

Marital status: Five studies assessed the impact of marital status on 30-day readmission. As shown in Table 3, the odds of being unmarried were significantly higher in patients who were readmitted compared to the patients who were not readmitted (OR: 1.52, 95% CI: 1.21-1.91, p-value: 0.003). High heterogeneity was reported among the study results (I-square: 86%).

Employment status: Four studies assessed the impact of employment status on 30-day readmission. As shown in Table 3, the odds of being unemployed were higher in patients who were readmitted compared to patients who were not readmitted, but the difference was statistically insignificant (OR: 1.21, 95% CI: 0.94-1.56, p-value: 0.13). Moderate heterogeneity was reported among the study results (I-square: 54%).

Insurance status: Seven studies assessed the impact of insurance status on 30-day readmission. As shown in Table 3, the odds of being uninsured were significantly lower in patients who were readmitted compared to patients who were not readmitted (OR: 0.65, 95% CI: 0.53-0.80, p-value: 0.001). High heterogeneity was reported among the study results (I-square: 95%).

Age (years): A pooled analysis of six studies showed no significant difference in the mean age of patients who were readmitted and patients who were not readmitted (MD: -0.55, 95% CI: -2.50 to 1.39, p-value: 0.58), as shown in Table 3. High heterogeneity was reported among the study results (I-square: 99%).

Length of stay (days): Length of hospital stay was significantly lower in patients who were readmitted compared to their counterparts (MD: -1.18, 95% CI: -2.16, -0.19, p-value: 0.02) as shown in Table 3. High heterogeneity was reported among the study results (I-square: 86%).

Risk of Readmission in Psychological Illnesses

**Figure 2:**
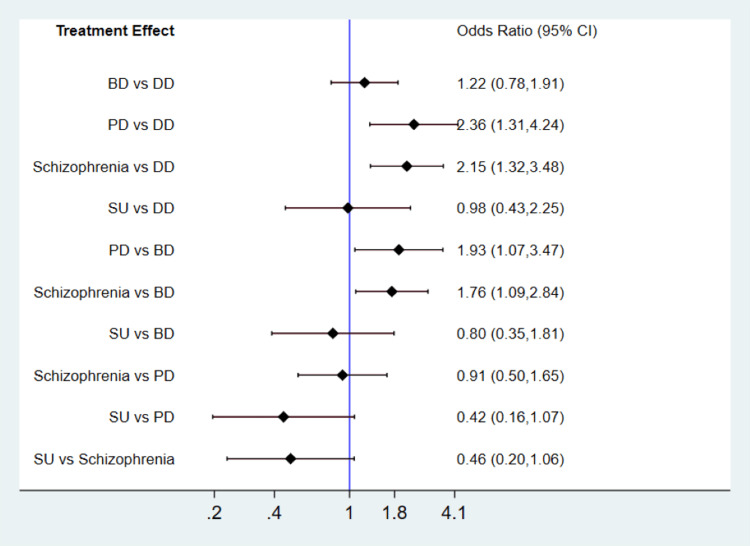
Pairwise comparisons of 30-day readmission among different psychiatric illnesses BD: bipolar disorder; DD: depressive disorder; SU: substance abuse; PD: personality disorder; CI: confidence interval

We compared rates of 30-day readmission among five illnesses including depressive disorder, bipolar disorder, substance abuse, schizophrenia, and personality disorder. The pairwise comparisons are shown in Figure 2. Overall, the rate of 30-day readmission was greater in patients with primary diagnosis of schizophrenia followed by personality disorder, bipolar disorder and depressive disorder. The lowest rate of 30-days readmission was reported in patients with primary diagnosis of substance abuse.

Discussion

This meta-analysis included 13 studies on risk factors associated with 30-day unplanned hospital readmission in the acute mental health setting. The pooled rate of 30-day hospital readmission was 16% (95% CI: 13%-20%). The pooled analysis showed that factors significantly associated with unplanned hospital readmissions included gender, length of stay, and land insurance status as predictors of unplanned hospital readmissions among individuals with psychiatric illnesses. Additionally, we also found that the rate of 30-day unplanned admissions was greater in patients with schizophrenia, followed by personality disorder, bipolar disorder, depression, and substance use.

We found that being male was one of the risk factors for hospital readmission. The majority of included studies reported a greater number of males among patients who were readmitted. Additionally, no significant association was reported between age and readmission. The current meta-analysis did not report any impact of employment status on the rate of readmission, and the results were consistent across the studies.

The present meta-analysis also reported that being unmarried was associated with readmissions. This is significant because marital status could suggest the practical role of a support system after discharge [31]. The presence of support can also enhance treatment adherence. Studies have found that married individuals are more likely to adhere to treatment plans, including taking medications as prescribed and attending follow-up appointments [32].

Findings also indicate that having insurance was associated with 30-day readmissions. There could be several factors contributing to this association. One possible explanation is that individuals with insurance have better access to mental health services and resources. With insurance coverage, they may have greater opportunities to seek treatment and follow-up care, which could increase the likelihood of readmission within a shorter timeframe [33]. Another factor to consider is the availability and quality of outpatient care. Patients with insurance coverage may have more options for outpatient services, such as therapy or medication management, which could result in a higher chance of readmission [34]. In some cases, inadequate or insufficient outpatient care may lead to relapse or worsening of symptoms, necessitating readmission to a psychiatric facility.

Length of stay was another significant predictor of 30-day readmissions. Extensive research has consistently shown the association between length of stay and readmission rates in psychiatric settings. A shorter length of stay may not provide sufficient time for effective stabilization and treatment during the initial hospitalization [35]. Psychiatric disorders often require a comprehensive assessment, medication adjustments, therapy, and discharge planning, and if the length of stay is insufficient, individuals may be discharged prematurely without achieving optimal stabilization, increasing the risk of relapse and subsequent readmission [36].

The present meta-analysis reported a high risk of readmission among patients with schizophrenia. Patients diagnosed with schizophrenia face an elevated risk of readmission, which can be attributed to their limited understanding of their illness. As a result, they may have a reduced awareness of impending worsening symptoms and the need for timely intervention [37]. Additionally, due to the nature of their symptoms, they might distance themselves from individuals who could otherwise support them in seeking care during a deterioration of their condition. Consequently, these patients may require an extended duration to achieve stabilization, both during their hospitalization and in the outpatient setting [26].

Our study reported a higher rate of readmission in patients with personality disorders. The presence of impulsive behavior, intense mood swings, and suicidal thoughts are characteristic features of personality disorders, leading to the presumption that a significant number of these patients are admitted to mental health units. Earlier studies have reported noteworthy findings regarding the readmission rates within 30 days for individuals diagnosed with borderline personality disorder [38-39].

The current meta-analysis also had certain limitations. The first limitation is the potential geographic bias due to the majority of included studies having been conducted in the United States. The over-representation of studies from a single country may limit the generalizability of the findings to other cultural contexts or healthcare systems. Second, certain factors were not assessed by the majority of studies, including treatment received and housing status. As these are important factors that can affect psychiatric readmission, understanding the impact of these factors is important in developing interventions. Therefore, in the future, more studies should be conducted assessing hospital-related factors along with demographic characteristics.

## Conclusions

In conclusion, this meta-analysis provides valuable insights into the risk factors associated with 30-day unplanned hospital readmissions in the acute mental health setting. The findings indicate that several factors significantly predict readmission rates among individuals with psychiatric illnesses. The findings indicate that gender, marital status, insurance status and length of stay significantly contribute to the likelihood of readmission. The analysis also revealed that patients diagnosed with schizophrenia had the highest rate of 30-day readmission, followed by personality disorder, bipolar disorder, depression, and substance use. This highlights the importance of providing targeted interventions and support for individuals with these conditions to reduce the risk of readmission. Future studies should focus on assessing hospital-related factors in conjunction with demographic characteristics to provide a more comprehensive understanding of the factors influencing readmission rates.
